# Participation of Semaphorin Family and Plexins in the Clinical Course of Patients with Inflammatory Bowel Disease

**DOI:** 10.3390/ijms252212442

**Published:** 2024-11-19

**Authors:** Gabriela Fonseca-Camarillo, Janette Furuzawa-Carballeda, Diana Aguilar-León, Braulio Martínez-Benítez, Rafael Barreto-Zúñiga, Jesús K. Yamamoto-Furusho

**Affiliations:** 1 Inflammatory Bowel Disease Clinic, Department of Gastroenterology, Instituto Nacional de Ciencias Médicas y Nutrición Salvador Zubirán, Mexico City CP 14080, Mexico; gabrielafaster@gmail.com; 2 Department of Immunology, Instituto Nacional de Cardiología, Ignacio Chávez, Mexico City CP 14080, Mexico; 3 Department of Experimental Surgery, Instituto Nacional de Ciencias Médicas y Nutrición Salvador Zubirán, Mexico City CP 14080, Mexico; jfuruzawa@gmail.com; 4 Medicine School, Universidad Panamericana, Mexico City CP 0390, Mexico; 5 Department of Pathology, Instituto Nacional de Ciencias Médicas y Nutrición Salvador Zubirán, Mexico City CP 14080, Mexico; aguilarleon@hotmail.com (D.A.-L.); brauliomb77@yahoo.com.mx (B.M.-B.); 6 Department of Endoscopy, Instituto Nacional de Ciencias Médicas y Nutrición Salvador Zubirán, Mexico City CP 14080, Mexico; barretozu@yahoo.com

**Keywords:** semaphorins, plexins, IBD

## Abstract

Semaphorins are an immunoregulatory protein family. Plexins bind semaphorins (SEMAs) and can form receptor complexes that give them chemotactic capacity. The role and expression profile of semaphorins and plexins in inflammatory bowel disease (IBD) is currently unknown. Aim: Characterize the semaphorins and plexins gene and protein expression in intestinal tissue from IBD patients and correlate them with the clinical phenotype. Material and Methods: This comparative and cross-sectional study enrolled 54 diagnosed IBD patients and 20 controls. Gene and protein expression of semaphorins and plexins were determined by RT-PCR and IHQ for the co-localization with neutrophils (myeloperoxidase, MPO) or CD123 plasmacytoid dendritic cells in intestinal tissue from IBD patients. Results: Colonic mucosa from active and remission ulcerative colitis (UC) had a significantly lower *SEMA4D* and *PLXNA1*, but higher *PLXNB1* gene expression than the control group. The only significant difference between active UC and remission was observed in the higher gene expression of *SEMA6D* in remission. It was associated with histological remission (*p* = 0.01, OR = 15, 95% CI: 1.39–16.1). The low expression of *PLXNA1* was associated with mild intermittent activity with two relapses per year (*p* = 0.003, OR = 0.05, CI = 0.006–0.51). Higher SEMA4D+ positive cells were detected in the submucosa, while PLXNC1+/MPO+ in the mucosal and submucosa of active UC patients compared with controls. Conclusions: The increased expression of the semaphorin and plexin family in IBD patients suggests their immunoregulatory function and is associated with remission and clinical phenotype in patients with UC.

## 1. Introduction

Ulcerative colitis (UC) and Crohn’s Disease (CD) are pathologies characterized by inflammation of the intestinal tract, with remission and recurrence periods of variable frequency. Although the etiology of both entities is still not completely understood, the most accepted theory about their origin is based on the complicated interaction between environmental factors and an inappropriate immune response that occurs in genetically susceptible individuals [1]. In the physiopathology of inflammatory bowel disease (IBD), alterations in molecules that regulate the inflammation response have been identified [2].

The different molecules that participate in the immunobiology of IBD are fundamental for developing this disease (adhesion molecules, cytokines, chemokines, and their receptors). They are the main signs in this network of cellular interactions that seem to occur as a result of the incapacity of the organism to respond adequately to damage and other environmental factors [3].

Recently, the role in maintaining immune homeostasis of a family of proteins called “semaphorins” (SEMAs) has been reported, regulating and coordinating the different communication systems through lymphocyte trafficking [4]. Initially, these proteins were discovered and described in the nervous system [5].

More than twenty types of semaphorins have been described, and their functions include angiogenesis, vasculogenesis, osteoclastogenesis, and regulation of the immune response [4].

Semaphorins are structurally characterized by an extracellular amino-terminal domain highly conserved and denominated as the “sema” domain. Based on the similarity of the structure of their C-terminal structure and amino acid sequence, this diverse group of proteins is divided into the following eight subclasses:

- Membrane-associated (classes I, IV, and VII);
- Secreted (classes II, III, and VIII);
- Plexins and neuropilins (Nrps) have a role in their signaling and primary receptor complexes [6].

Membrane-associated semaphorins play an important role in the homeostasis of the immune response through the control of lymphocyte trafficking, IL-12 production derived from DC, downregulation of neutrophil activation, and intercellular communication to activate the immune system response [7].

Most membrane-associated semaphorins such as SEMA4D (CD100), SEMA6D, and SEMA7A use plexins as receptors, whereas soluble class III semaphorins (except SEMA3E that binds directly to plexin-D1) require neuropilins as co-receptors. However, studies have shown that semaphorins also signal independently of plexins through other receptors [6].

In the case of class VI semaphorins, also denominated SEMA4A and SEMA4D, they are constitutively expressed in T cells and dendritic cells and interact with immunoglobulins and mucin domains-containing protein 2 (TIM-2) and CD72 in T cells. [8]. Sema4D and its receptor CD72 are expressed in large amounts in T cells and DC, respectively. Sema4D-deficient DC exhibits reduced expression of co-stimulatory molecules and IL-12 cytokine production. In addition to T cells and DC, Sema4D is also expressed in neutrophils. Sema4D–plexinB2 interaction decreased NET formation and acted as a negative regulator of neutrophil activation [9].

SEMA6D is expressed in B cells, T cells, NK cells, and dendritic cells, while SEMA7A is expressed in T cells and shows a positive regulation of immune system function [10]. SEMA7A is recruited to the lipid rafts in the immunological synapse produced between T cells and macrophages, where it interacts with the α1β1 integrin and the extracellular matrix to hold effective cells like macrophages in the inflammation site. Therefore, SEMA7A seems to be implicated in the inflammation processes [6,7,8,9].

Some of these semaphorins are involved in the pathogenesis of autoimmune disorders. Recently, low expression of SEMA3A and SEMA7A has been reported in systemic lupus erythematosus [10]. However, no studies have been performed on these proteins in IBD.

This family of regulatory proteins may represent a new target for the treatment of UC and CD, and knowing about their participation is essential for future research and clarification of the origin and development of IBD.

The aim of this study was to characterize the semaphorins and plexins gene and protein expression in intestinal tissue from IBD patients and correlate them with the clinical phenotype.

## 2. Results

A total of 54 consecutive patients with a diagnosis of IBD confirmed by histopathology were included, of whom 34 had UC (17 active and 17 in remission), 20 with active CD, and 20 controls without intestinal inflammation. The clinical and demographic characteristics are shown in Table 1.

### 2.1. Gene Expression Levels of Semaphorins (SEMA4D, SEMA5A, and SEMA6D) in the Colonic Mucosa of Patients with IBD

**Figure 1:**
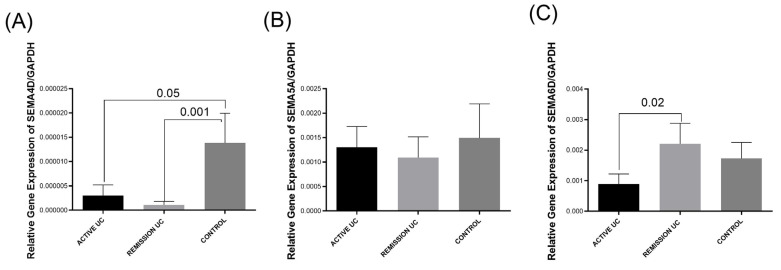
Semaphorins gene expression analysis in UC patients and controls. (**A**) *SEMA4D* gene expression, (**B**) *SEMA5A* gene expression, (**C**) *SEMA6D* gene expression. Bars show means ± S.E.M. of SEMAS transcript levels with *GAPDH* as the housekeeping gene. Differences among groups were assessed by the Kruskal–Wallis test. *p*-value < 0.05 was considered significant.

Gene expression levels of semaphorins *SEMA4D, SEMA5A*, and *SEMA6D* were analyzed in the rectal mucosa of patients with UC and ileum mucosa of CD patients (Figure 1). Relative gene expression of the *SEMA4D* was higher in the control group colonic mucosa compared with the active (*p* = 0.05) and remission UC group (*p* = 0.001; Figure 1A). No differences were determined between the study groups concerning the genetic expression of *SEMA5A* (Figure 1B). *SEMA6D* gene expression was higher in remission than in active UC patients (*p* = 0.02; Figure 1C). It was associated with histological remission (*p* = 0.01, OR = 15, 95% CI = 1.39–16.1).

### 2.2. Gene Expression of Plexin Receptors (PLXNA1, PLXNB1, PLXNB2, and PLXNC1) in the Colonic Mucosa of Patients with UC

**Figure 2:**
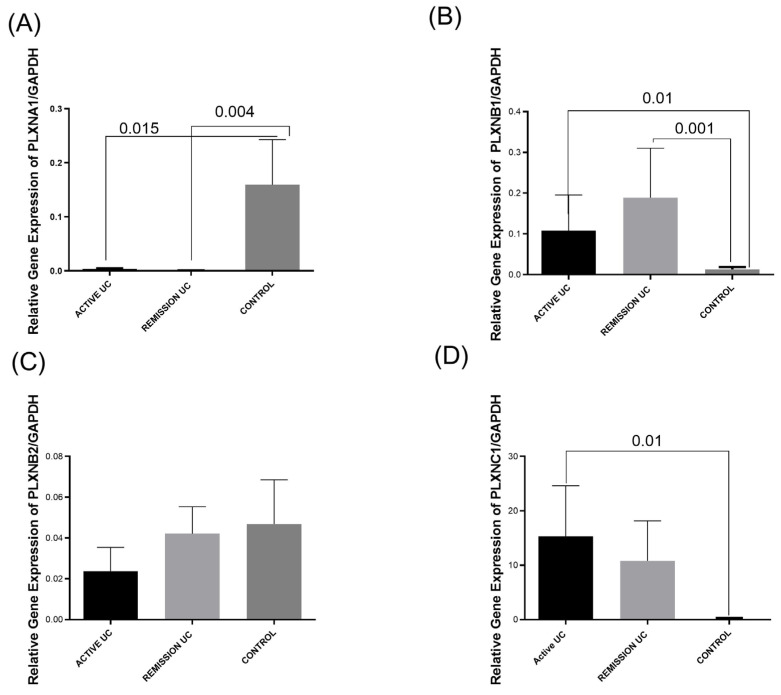
Plexins gene expression analysis in UC patients and controls. *p*-value < 0.05 was considered significant. (**A**) *Plexin A1* gene expression, (**B**) *plexin B1* gene expression, (**C**) *plexin B2* gene expression, (**D**) *Plexin C1* gene expression. Relative gene expression. Bars show means ± S.E.M. of plexins transcript levels with *GAPDH* as the housekeeping gene. Differences among groups were assessed by the Kruskal–Wallis test. *p*-value < 0.05 was considered significant.

*PLXNA1* mRNA levels were higher in the control group compared with the active UC group (*p* = 0.015) and remission UC group (*p* = 0.004; Figure 2A). We found a risk association where a low *PLXNA1* expression correlates with mild intermittent activity and two relapses per year (*p* = 0.003, OR = 0.05, 95%CI = 0.006–0.51).

For *PLXNB1* mRNA levels, we detected an increased gene expression in patients with UC remission (*p* = 0.001) and active UC patients (*p* = 0.01) compared with controls (Figure 2B). No differences were determined between the study groups concerning the genetic expression of *PLXNB2* (Figure 2C). *PLXNC1* gene expression was statistically significantly elevated in the active UC group compared to the controls (*p* = 0.01; Figure 2D).

### 2.3. Co-Localization of Semaphorin 4D and Plexins B1 and C1 in Intestinal Tissue of IBD Patients

Tissue samples were collected from 10 UC, 10 CD patients with severe activity (refractory to treatment), and 5 control patients (without data of intestinal inflammation). Histopathological analysis of the samples from patients with UC with severe activity showed architectural distortion, dense lymphoplasmacytic infiltrate from the mucosa to the serosa, distortion of the crypts, and pseudo-villous aspect of the colonic surface.

Patients with IBD exhibit extensive inflammatory infiltrates primarily composed of mononuclear cells extending from the serosal layer to the mucosa. These infiltrates are particularly abundant at the epithelial level. In all cases, significant mucosal destruction was observed in mononuclear cells at the sites of ulceration in the lamina propria.

**Figure 3:**
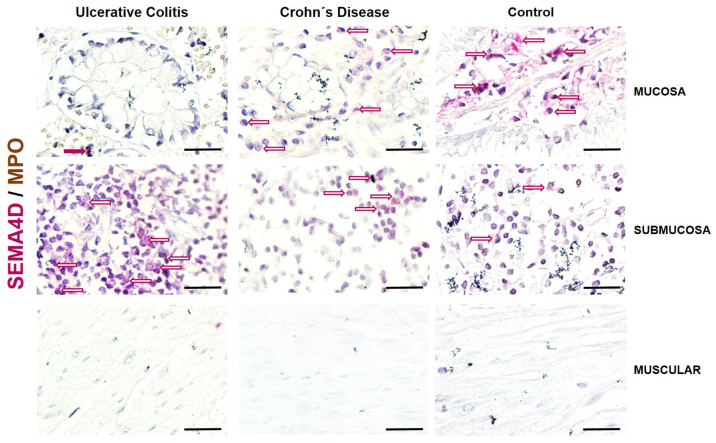
Representative image of SEMA4D/MPO detection and localization in intestinal tissue from UC and CD patients and controls without intestinal inflammation. SEMA4D-positive cells were detected in pink and MPO in brown. Burgundy arrows: Double positive cells; Red outlined arrows: SEMAD4 positive cells; Brown outlined arrows: MPO positive cells. The original magnification was 600X. (Scale bar = 50 µm).

Cells that expressed SEMA4D (pink-stained cells) presented a predominantly lymphoid-like phenotype. These were found distributed throughout the submucosa and predominantly in patients with active UC. No MPO+ cells (neutrophils; brown-stained cells) were determined to co-express SEMA4D (Figure 3).

**Figure 4:**
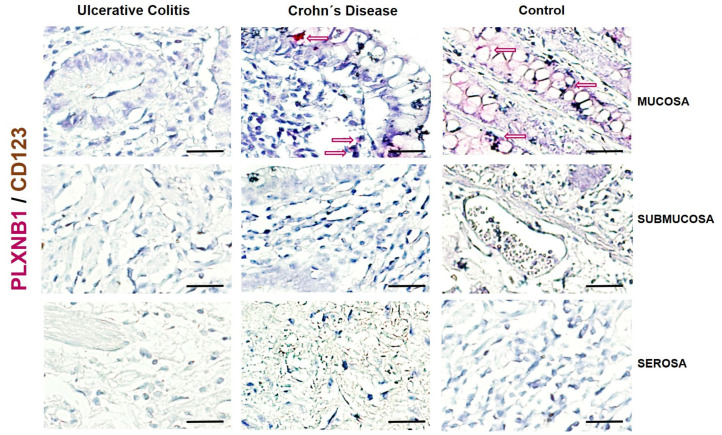
Representative image of PLXNB1/MPO detection and localization in intestinal tissue from UC and CD patients and controls without intestinal inflammation. PLXNB1-positive cells were detected in pink and MPO in brown. Burgundy arrows: Double positive cells; Red outlined arrows: PLXNB1 positive cells; Brown outlined arrows: CD123 positive cells. The original magnification was 600X. (Scale bar = 50 µm).

The protein expression of PLXNB1 (pink-stained cells) was practically undetectable in patients with UC and CD (Figure 4). No MPO+ cells (neutrophils; brown-stained cells) were determined that co-expressed PLXNB1.

**Figure 5:**
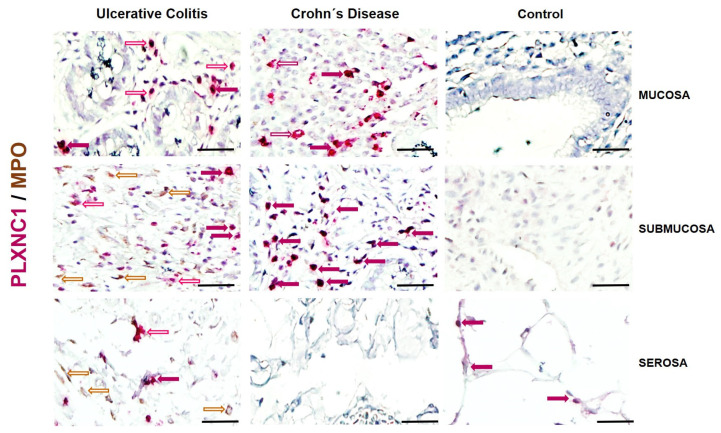
Representative image of PLXNC1/MPO detection and localization in intestinal tissue from UC and CD patients and controls without intestinal inflammation. PLXNC1-positive cells were detected in pink and MPO in brown. Burgundy arrows: Double positive cells; Red outlined arrows: PLXNC1 positive cells; Brown outlined arrows: MPO positive cells. The original magnification was 600X. (Scale bar = 50 µm).

The synthesis of plexin C1-positive cells, co-localizing with MPO was analyzed in the same three tissue layers. The PLXC1/MPO double-positive cells were primarily located in the mucosal epithelial cells and the submucosa in the inflammatory infiltrate of the lamina propria and submucosa of individuals with active UC compared to those with active CD and controls (Figure 5).

## 3. Discussion

This is the first study that demonstrates the expression of immune semaphorins and their receptors (plexins) in the intestinal tissue of IBD patients in different clinical stages (activity and remission) and their association with the phenotype and clinical course of the disease, as well as the potential use of these molecules as therapeutic targets for the treatment of IBD.

In the present study, we reported that *SEMA4D* and *SEMA6D* gene expression was decreased in the rectal mucosal biopsies from active UC patients compared to the control individuals, suggesting that they perform a negative immune regulation role in colonic mucosal inflammation. This is consistent with findings in murine models of colitis where decreased expression of *SEMA4D* significantly aggravated the disease [11] with increased colonic ulceration and mucosal inflammatory cell infiltration. Similarly, the immunoregulatory activity of SEMA6D has prevented the development of colitis, and its inhibition has exacerbated it [12]. However, semaphorins of the same family may mediate inflammation in UC and CD distinctly since a study in patients with UC and CD found higher expression of a class IV semaphorin in colon biopsies compared to control groups, proposing blocking activity only in active forms of the disease [13].

There is evidence of the anti-inflammatory activity of SEMA6D mediating M2 macrophage polarization in mice, and its deficiency predisposes colitis development [12]. The above supports our findings because there is an increase in *SEMA6D* expression in the UC group in remission concerning the active UC group (*p* = 0.02), and high *SEMA6D* gene expression was associated with histological remission (*p* = 0.01, OR = 15, 95% CI = 1.39–16.1), confirming the involvement of SEMA6D in the regulation of the intestinal inflammatory response and its impact on the clinical phenotype of the disease.

Conversely, we found a higher expression of plexins like *PLXNA1* and *PLXNB2* in the control group, which was demonstrated in the difference with the UC groups (active and remission) who presented low expression at the mRNA level. Previously published reports showed that PLXNA1 functions as a receptor for SEMA6D [14], while PLXNB2 functions as a high-affinity receptor for SEMA4D [11]. In addition to this, lower expression of these two plexins in the colon would generate a greater predisposition to inflammation. PLXNB2 actively participates in the epithelial repair of the colon together with SEMA4D [11,15]. For this reason, an increase in the expression of this receptor was observed in patients in remission of UC concerning active cases.

An interesting finding was that decreased *PLXNA1* levels are associated with mild intermittent activity with two relapses per year in UC; the downregulation of expression of this receptor seems to impact the clinical course in the patients and predispose relapses and maintenance of UC activity.

In control patients, SEMA4D+ cells were predominantly localized in the submucosal layer, where the primary source seems to be lymphoid cells. In contrast, in patients with active UC, only a few double-positive cells were in the inflammatory infiltrate of the submucosa, while in CD patients, only a small number of positive cells were in the mucosa. Differential expression of semaphorins and plexins in mucosal intestinal biopsies from IBD patients is suggestive of its central role in regulating local tissue inflammation in the bowel.

A mouse model of colitis showed a poor response to epithelial damage in the absence of SEMA4D maintaining myeloperoxidase activity, suggesting a selective defect in the response of infiltrating lymphocytes [11].

The direct participation of classes III and IV (SEMA4D) in epithelial and crypt cells in repairing inflamed and damaged colonic epithelium has been demonstrated in vivo [11,16]. Consistent with this, the control group of this work had as the primary source of SEMA4D the epithelial cells, goblet cells, and crypt cells of the mucosa and submucosa, confirming that the absence or poor production at the level of mRNA and subsequent low synthesis of this protein has an impact on the development and clinical course of ulcerative colitis.

These findings correlate with a previous report of differential expression of the percentage of T regulatory cells expressing SEMA3A in patients with active CD and active UC, which was significantly lower when compared to that of the healthy controls (*p* < 0.001 and *p* < 0.0001, respectively), where the authors suggest that this is partially responsible for their failure in preventing CD4+ effector T cell-induced inflammation in IBD in peripheral blood [16].

In addition, our findings correlate with the lack of expression and synthesis of PLXNC1 at the tissue level in mucosal, submucosal, muscular, and serosa layers of patients with active UC and CD. Only positive cells that were morphologically compatible populations of neutrophils, NK cells, and infiltrating macrophages were detected in the submucosal area of intestinal tissue of patients with active UC compared to the CD group and controls.

All these results and observations suggest that an increased expression of the semaphorins family, SEMA4D for IBD patients in remission, has immunoregulatory and anti-inflammatory functions. In contrast, low PLXNA1 expression is associated with continuous activity in patients with UC during their clinical evolution. It seems that a pattern is present in the peripheral inflammatory response in patients with these diagnoses and not in others that also affect the colon [12]. Therefore, further studies on the semaphoring-mediated crosstalk between immune cells and intestinal epithelial cells are required to understand this scenario better. Much remains to be learned, however, about the pathogenic mechanisms that lead to IBD.

It is essential to mention that no previous studies have analyzed the characterization of these proteins and their impact on the clinical phenotype of IBD patients.

## 4. Materials and Methods

### 4.1. Patients and Methods

A comparative cross-sectional study included 34 patients with UC (17 with activity and 17 in remission) and 20 patients with active Crohn’s Disease. The control group included 17 patients without clinical and endoscopic data of intestinal inflammation or systemic disease (cancer, autoimmune diseases, diverticular disease, drug-induced colitis, post-radiation colitis, infectious colitis, and ischemic colitis). All patients were recruited from the Inflammatory Bowel Disease Clinic from the Department of Gastroenterology at the Instituto Nacional de Ciencias Médicas y Nutrición, Salvador Zubirán. Only patients who signed the informed consent were included in the study. The control group consisted of patients on medical weight loss and anemia assessment at the same reference center. The disease activity in UC patients was evaluated by the novel integral disease activity index or Yamamoto-Furusho index, which includes clinical, biochemical, endoscopic, and histological findings [17].

A total of 71 rectal mucosa biopsies were collected to quantify the gene expression of members of the semaphorins family (SEMA4D, SEMA5A, SEMA6D, and SEMA7A) and their receptors (PLXNA1, PLXNB1, PLXNB2, and PLXNC1). The intestine biopsies were placed in cryovial tubes with 1 mL of tissue storage reagent (RNA later^®^) and stored at -70 °C until the RNA extraction.

### 4.2. RNA Total Extraction and Reverse Transcription-PCR

Relative mRNA expression analysis was performed using the previous standardized methodology [18,19,20,21]. Total RNA was extracted from the mucosal rectal biopsies utilizing an RNA extraction kit according to the methodology suggested by the manufacturer. The biopsies were mixed with the homogenizer for 1 min in lysis buffer and washed with 100% ethanol using purification columns. The mixture was centrifuged at 13,000× *g* for 15 s and then washed with a buffer at 13,000× *g* for 15 s. Finally, 100 μL of elution buffer was added to dilute the total ARN. An aliquot of each RNA product was electrophoresed on a 1% agarose gel, visualized by staining with ethidium bromide, and documented with a transilluminator with ultraviolet light.

Complementary strand DNA was synthesized from the total RNA by Reverse Transcription. The reaction will be carried out with 20 μL as follows: preincubation—25 °C × 10 min, incubation—55 °C × 30 min, followed by denaturation—85 °C × 5 min in a thermocycler (Perkin-Elmer, Waltham, MA, USA). The polymerase chain reaction was performed using the complementary DNA that resulted from retrotranscription as a substrate. For amplification of regions of interest, the reaction was performed in 10 μL under the following conditions: a denaturation program at 95 °C for 10 min, 45 amplification cycles (95 °C for 10 s, alignment 60 °C for 10 s, extension 40 °C for 30 s), and a cooling cycle at 40 °C for 30 s. To determine the relative expression of the target genes and *GAPDH* (Glyceraldehyde Phosphate Dehydrogenase, as a reference gene), the Roche ^®^ Light Cycler 480 Thermocycler (Roche, Vienna, Austria) was used using validated assays for quantification (reproducibility and linearity), with sense and antisense primers from Invitrogen^®^ and Taq Man probes for each gene (Universal Probe Library Set, Human by Roche ^®,^ Vienna, Austria), as shown in Table 2. For qPCR assay quality control, the determination of linearity and reproducibility was evaluated (VC < 10%). The mRNA relative quantification of target genes was conducted using the LightCycler software 4.1. Gene expression values were normalized to the value of the housekeeping gene *GAPDH* and calculated based on the comparative cycle threshold Ct method (2-ΔΔCt). The relative gene expression levels of the semaphorins family and plexins were normalized. For target genes, we employed CT values cycles (Ct)* high (>24–35) and reference gene GAPDH CT (20–23).

### 4.3. Detection of SEMA4D and Plexins Members (PLXNB1 and PLXNC1) by Immunohistochemistry in Intestinal Tissue

To identify cells expressing SEMA4D, PLXNB1, and PLXNC1, 4 µm thick paraffin-embedded tissue sections were placed on an electrocharged slide. We followed the methodology of the double-staining procedure [18,19,20,21]. The slides were dewaxed for 45 min at 54 °C. The tissue was hydrated with xylene, alcohol (100%, 70%, 50%), and distilled water. Then, heat-mediated antigen retrieval with DIVA solution (Biocare Medical, Pacheco, CA, USA) was performed. Subsequently, the slides were incubated with a solution of 3% H_2_O_2_ in absolute methanol (1:9 vol/vol) for 20 min to eliminate the activity of endogenous peroxidase, pseudo-peroxidase, and alkaline phosphatase. Nonspecific staining was avoided by incubating the tissues with a sniper-blocking solution (Biocare Medical, Pacheco, CA, USA) for 20 min. Tissues were incubated for 30 min at room temperature with rabbit polyclonal anti-MPO/anti-mouse SEMA4D, anti-MPO/mouse anti-PLXNC1, or mouse anti-PLXNB1 (Santa Cruz Biotechnology, Santa Cruz, CA, USA) at a concentration of 10 µg/mL. Slides were washed and then incubated with PolyView IHCh reagent (mouse-HRP) and PolyView IHCh reagent (rabbit-AP) for 20 min (MultiView (mouse-HRP/rabbit-AP) Enzo Life Sciences, Farmingdale, NY, USA). Finally, antigens were visualized using horseradish peroxidase (HRP)/3,3′-diaminobenzidine (DAB), and the second antigen with alkaline phosphatase (AP)/Permanent Red. Tissues were counterstained with Mayer’s hematoxylin and contrasted with a 37 mmol/L ammonium hydroxide solution, after which they were left to dry at room temperature and mounted in resin. Regardless of staining intensity, all stained cells were evaluated by estimating each tissue’s relative percentage of negative cells in four random fields (320×). Negative control stains were performed with normal human serum diluted 1:100, in place of the primary antibody, and the negative universal control reagent designed explicitly for rabbit, mouse, and goat antibodies (universal negative control reagent, IHC, Enzo Life Sciences). The reagent blank was incubated with phosphate-buffered saline and egg albumin (Sigma-Aldrich, Saint Louis, MO, USA) instead of the primary antibody. All controls excluded nonspecific staining or endogenous enzyme activities.

### 4.4. Statical Analyses

Data were analyzed with the SPSS v22 statistic package. Quantitative variables were compared using the Mann–Whitney U test for independent samples and the Kruskal–Wallis–Dunn test for multiple comparisons. Categorical variables were analyzed using Pearson’s chi-squared test and Fisher’s exact chi-squared test for values less than 5. Association strength was determined using an odds ratio > 1, which was considered positive or indicative of susceptibility, and <1 indicates negative or protection. The Spearman correlations test was used to analyze the association of gene expression of SEMA4D, SEMA5A, SEMA6D, SEMA7A PLXNA1, PLXNB1, PLXNB2, and PLXNC1 with clinical variables. Statistical significance was determined with a *p*-value < 0.05.

## 5. Conclusions

The increased expression of the semaphorin and plexin family in IBD patients suggests their immunoregulatory function and is associated with remission and clinical phenotype in patients with UC. Semaphorins are molecules that regulate and coordinate different immune cell communication systems, helping to maintain immune homeostasis. For this, they seem to be involved in pathologies associated with the immune system. This family of proteins could be used as new alternatives in treating IBD.

## Figures and Tables

**Table 1:** Clinical and demographic characteristics of IBD patients.

	Active Ulcerative Colitis (*N* = 17)	Remission Ulcerative Colitis (*N* = 17)	Active Crohn’s Disease (*N* = 20)	Controls (*N* = 20)
Average age	43.29	42.8	41.75	45.7
Gender Female/male	12/5	10/6	12/8	7/13
Clinical Course				
Initial active	3/17	14/17	1/20	
Mild intermittent activity <relapse/year	13/17	3/17	5/20	
Continuous activity (treatment–refractory)	1/17	0/17	14/20	
Extent of Disease				
Pancolitis (E1)	14/17	9/17		
Proctosigmoiditis (E2)	2/17	1/17		
Left-sided colitis (E3)	1/17	7/17		
Extra-intestinal Manifestations				
Presents	7/17	4/17	18/20	
Absent	10/17	13/17	2/20	
Treatment Response				
Favorable	12/17	17/17	0/20	
No response	5/17	0/17	20/20	

**Table 2:** Characteristics of the primers used in real-time PCR.

Gene	NM Gene Bank	Forward Primmer	Reverse Primmer	Universal Probe Library Set
*SEMA4D*	NM_001142287.1, NM_006378.3	aagtgggtgcgctataatgg	tcaaggagctggtgtagttgg	Probe # 1
*SEMA5A*	NM_003966.2	cccctgaatcagacctcaag	cctctcagggaagagctcaa	Probe # 11
*SEMA6D*	NM_001198999.1, NM_153619.1, NM_153617.1, NM_020858.1, NM_153616.1, NM_153618.1, NM_024966.2	gtcgaacataataatttaggcaagg	acagttcagccgagccttta	Probe # 47
*SEMA7A*	NM_001146029.2, NM_001146030.2, NM_003612.4	cctttcatgtgctttacctaactaca	gatgttgaaggcgaagctgt	Probe # 1
*PLXNA1*	NM_032242.3	cagtgatgtggctgtgtcg	tcaggcaccggatctcac	Probe #2
*PLXNB1*	NM_002673.5, NM_001130082.2	aggtactatgcagacatcagacaga	aggtctccggagtagttccag	Probe # 20
*PLXNB2*	NM_012401.3	ggccgtcatctgcaactc	taaggaggagctggatggtc	Probe # 89
*PLXNC1*	NM_005761.2	cctcttcagaatgagtgagatcc	caggttgatccccttctttg	Probe #33
*GAPDH*	NM_002046.3	agccacatcgctcagacac	gcccaatacgaccaaatcc	Probe # 60

## Data Availability

Research data are available upon reasonable request.
